# Individual, institutional, and scientific environment factors associated with questionable research practices in the reporting of messages and conclusions in scientific health services research publications

**DOI:** 10.1186/s12913-020-05624-5

**Published:** 2020-09-03

**Authors:** Reinie G. Gerrits, Joko Mulyanto, Joost D. Wammes, Michael J. van den Berg, Niek S. Klazinga, Dionne S. Kringos

**Affiliations:** Department of Public Health, Amsterdam UMC, University of Amsterdam, Amsterdam Public Health research institute, P.O. Box 22660, 1100 DD Amsterdam, The Netherlands

**Keywords:** Questionable research practices, Scientific reporting, Health services research, Reporting guidelines, Reporting checklist

## Abstract

**Background:**

Health Services Research findings (HSR) reported in scientific publications may become part of the decision-making process on healthcare. This study aimed to explore associations between researcher’s individual, institutional, and scientific environment factors and the occurrence of questionable research practices (QRPs) in the reporting of messages and conclusions in scientific HSR publications.

**Methods:**

We employed a mixed-methods study design. We identified factors possibly contributing to QRPs in the reporting of messages and conclusions through a literature review, 14 semi-structured interviews with HSR institutional leaders, and 13 focus-groups amongst researchers. A survey corresponding with these factors was developed and shared with 172 authors of 116 scientific HSR publications produced by Dutch research institutes in 2016. We assessed the included publications for the occurrence of QRPs. An exploratory factor analysis was conducted to identify factors within individual, institutional, and environmental domains. Next, we conducted bivariate analyses using simple Poisson regression to explore factors’ association with the number of QRPs in the assessed HSR publications. Factors related to QRPs with a *p*-value < .30 were included in four multivariate models tested through a multiple Poisson regression.

**Results:**

In total, 78 (45%) participants completed the survey (51.3% first authors and 48.7% last authors). Twelve factors were included in the multivariate analyses. In all four multivariate models, a higher score of “pressure to create societal impact” (Exp B = 1.28, 95% CI [1.11, 1.47]), was associated with higher number of QRPs. Higher scores on “specific training” (Exp B = 0.85, 95% CI [0.77–0.94]) and “co-author conflict of interest” (Exp B = 0.85, 95% CI [0.75–0.97]) factors were associated with a lower number of QRPs. Stratification between first and last authors indicated different factors were related to the occurrence of QRPs for these groups.

**Conclusion:**

Experienced pressure to create societal impact is associated with more QRPs in the reporting of messages and conclusions in HSR publications. Specific training in reporting messages and conclusions and awareness of co-author conflict of interests are related to fewer QRPs. Our results should stimulate awareness within the field of HSR internationally on opportunities to better support reporting in scientific HSR publications.

## Background

In 2009, it was estimated that 85% of research funding in biomedical sciences was avoidably wasted [1]. In the biomedical sciences, evidence has been piling up on questionable research practices (QRPs) such as imbalanced research question selection, poor study design and execution, non-publication, and poor reporting [1]. Over time, advancements have been made to address these QRPs, including scientific reporting [2]. However, proper interpretation and reporting of messages and conclusions across different research methodologies in scientific publications requires more attention [3]. Researchers can introduce various QRPs in the reporting of messages and conclusions in their scientific publications (e.g., generalizing findings to populations not included in the study, not reporting contradictory evidence, claiming an unjustified causal relationship, and inadequately justifying conclusions) [3–5]. Moreover, although scientific reporting of biomedical studies is progressing [2], responsible scientific reporting requires greater awareness.

In this study we focus on the field of Health Services Research (HSR). HSR has a direct link to policy and practice, where stakeholders and funders may contribute considerably to the interpretation of results [6, 7]. Additionally, HSR relies on mixed methodologies such as qualitative and mixed methods designs that may have less strict reporting requirements compared to quantitative designs such as randomized controlled trials [3].

Recent work has suggested that scientific HSR publications may include a median of six QRPs in the reporting of messages and conclusions [8]. QRPs were primarily found in reported implications and recommendations for policy and practice, in a lack of mentioning of contradictory evidence, and in the conclusions of the scientific publication [8]. The occurrence of these QRPs is concerning as messages and conclusions reported in scientific HSR literature are often transferred to policy makers, managers, and the general public. Further, these groups may learn about messages and conclusions directly from the scientific publication or through societal publications such as professional journals, factsheets, press releases, and reports [7, 9–12]. Whether messages are disseminated by researchers, science communicators, or journalists, they may be accepted as established evidence and become part of the decision-making process on health and healthcare. Decisions on topics such as co-payments, adaption of protocols in hospitals, admitting medications to insurance packages, and tobacco regulation may thus be affected by inadequately reported messages and conclusions [9, 13, 14].

Scientific journals have taken the lead in implementing control measures to provide structure to the review process and improve responsible reporting [9]. These efforts have resulted in practices such as publication checklists [15], data sharing, open access [16] and public peer-review [17] becoming increasingly common. Yet, these measures are primarily aimed at increasing transparency in reporting and thus may be insufficient in preventing QRPs in the reporting of messages and conclusions specifically. To strengthen the reporting of messages and conclusions, measures may need to be taken at multiple levels, including academic journals and research institutions themselves [18]. QRPs may be caused by adverse incentives on institutional level, such as an inadequate reward system, lacking reporting infrastructures, and insufficient prepublication review [1]. Recently, Dutch academic and non-academic HSR institutions have begun to collaborate with the goal to increase responsible reporting of HSR findings. Non-academic institutions are research institutions independent of universities. These efforts have been supported by the Netherlands Organization for Health Research and Development (ZonMw).

HSR institutions in the Netherlands have varying organizational policies in fostering responsible conduct of research, including responsible reporting. This variety in institutional culture and organisation offers the opportunity to learn from each other’s reporting practices. Improving scientific publication of HSR requires an understanding of factors that influence authors in their writing, as well as those that impact the publication process itself (e.g. pressure and relationships with funders) [19–21]. Research institutions may prevent the occurrence of QRPs by improving internal integrity and training researchers in scientific writing and communication [19–21]. However, considering the specific characteristics of HSR, additional evidence is needed on how possible factors may relate to QRPs in messages and conclusions specifically [22].

Consequently, the aim of this study was to explore associations between individual, institutional, and scientific environment factors and the frequency of inadequacies in the reporting of messages and conclusions in scientific HSR publications.

## Methods

### Design

We employed a mixed-methods study design. First, we identified factors possibly contributing to the occurrence of QRPs in the reporting of messages and conclusions in scientific HSR publications through a literature review, 14 semi-structured interviews with leaders of HSR groups or institutions in the Netherlands, and 13 focus-groups amongst junior health services researchers. Factors were clustered into three domains: individual, institutional and scientific environmental domains [9]. Second, a survey corresponding to the identified factors was developed and shared with 172 first and last authors of a sample of 116 scientific HSR publications published in 2016 with an affiliation to Dutch HSR groups or institutions.

### Setting

The study involved publications and participants from 13 HSR groups, departments, or institutions including both academic and non-academic institutions (hereafter referred to as “HSR institutions”) in the Netherlands. These institutions agreed to participate in an effort to assure the overall quality of HSR publications in the Netherlands.

### Conceptual framework on factors potentially associated with QRPs in the reporting of messages and conclusions in HSR

Factors potentially associated with QRPs in HSR were identified through an exploratory literature review, 14 semi-structured interviews with 19 leaders/representatives of the 13 participating institutions, and 13 focus-groups comprised of 57 junior/PhD researchers at participating HSR institutions. An initial overview of factors was created through the literature review. This overview was then discussed in the semi-structured interviews with the leaders/representatives of participating institutions. Within the focus-groups, an open conversation was held with participants to identify additional factors overlooked in the literature and interviews (a focus group guide is provided as supplementary material 1). Documented interview reports and transcripts were qualitatively analysed in MaxQDA resulting in the specification of factors potentially associated with QRPs in the reporting of messages and conclusions in scientific HSR publications. The applied methods for the development of these factors are described in more detail in supplementary material 2.

Identified factors were included in a theoretical framework consisting of three domains: individual, institutional, and scientific environments. Of note, factors within each domain could be influenced by those in other domains. The individual domain was comprised of factors bound to the individual researcher, including those associated with research experience and self-efficacy. The institutional domain included factors controlled by the institution that houses the researcher. These included institutional culture, facilities, interactions, and policies that may affect the writing and publication experience of the researcher. More concretely, an institution may have an (unofficial) policy to produce a certain number of publications per year. The scientific environmental domain included factors that manifest outside of the direct control of the institution, including those characterizing scientific culture and systems in general.

The framework of included factors is provided in Table 1.

**Table 1:** Framework and included factors

1 Research environment ‘culture’	2 Institutional conditions	3 Individual researcher characteristics
**Funding** • Funding rewards innovation & novelty • Demands of the funder **Valorisation of research outcomes** • Revenue model • Public media • Media pressure **Policies & practices scientific society** • Competition for research positions • Journal policies & practices • Peer review process • Pressure to publish ‘exciting’ articles **Collaborating partners** • Conflicts of interest **Research beneficiaries / stakeholders** • Usefulness: study designed without proper consideration of the value for e.g. patients	**Structural conditions/resources** • Education • Reward system / incentives • Presence and adherence to a Research code • Recruitment & selection researchers • Presence of formal quality policy • Transparency study materials/data **Social conditions** • Opportunities for peer-discussion • Presence of colloquia for article discussion • Review of pre-publication findings • Competitiveness **Role of supervisors** • Task perception • Workload • Social skills (in supervision, and collaboration) **Cultural conditions** • Ideology institute • Functioning of the ICT infrastructure • Open organisation culture	**Motivation** • Promotion • Respect from peers • Focus on short-term success • Ideology **Capabilities** • Research training in HSR • Writing skills • Training research integrity • Social skills • Self-efficacy (to stand up to pressure) **Working conditions** • Workload • Work pressure **Perceptions** • Self-perception • Perception of others **Personality traits** • Narcissism

### Survey development

The survey was designed based on the framework described above. For each identified factor in the preliminary framework, one or more survey questions were developed. Questions were evaluated on their face validity by the co-authors and two project advisors, both senior health services researchers. For this study, we developed a new questionnaire, as no validated questionnaires were tailored to the field of HSR or scientific reporting. One existing question from the publication pressure questionnaire was included in our newly developed questionnaire [21]. The questionnaire was developed in English (i.e, the primary working language of the study population).

A “think out loud” test was performed with two people from the target population. RG sat down with two researchers individually as they answered the survey questions and commented on their interpretation. The survey was designed in English and checked by a native speaker. After the final revision, the survey included 97 questions related to the factors within individual, institutional and scientific environment domains. Seven additional questions were included to assess personal and background characteristics. Answers to survey questions were provided on a Likert-scale (strongly disagree, disagree, neither agree nor disagree, agree, and strongly agree).

The survey is provided in supplementary material 3.

### Survey study population

In a previous study we assessed QRPs in the reporting of messages and conclusions in 116 international peer-reviewed publications authored by researchers from the 13 participating institutions. QRPs were defined as “to report, either intentionally or unintentionally, conclusions or messages that may lead to incorrect inferences and do not accurately reflect the objectives, the methodology, or the results of the study.” [8] For the assessment, we used a detailed assessment form including 35 possible QRPs in reporting messages and conclusions (e.g., “conclusions that do not adequately reflect the findings of the study”, “limitations are not adequately justified”). This assessment form, along with corresponding methods and results, have been published elsewhere [6]. For the current study, we conducted a survey amongst the 172 first and last authors of these publications.

First and last authors of the 116 scientific publications were included in our assessment.

We identified a total of 202 authors (116 first authors and 86 unique last authors) as the sample for our study. Contact information (i.e., e-mail addresses) was obtained through the participating institutions. These institutions were asked to encourage their researchers to participate in the survey, however, participation was voluntary and participants could stop at any time. Participants of the survey were informed of the goal of the study and data handling procedures in the invitation e-mail and at the start of the survey. We excluded 30 authors who’s contact information was unknown, resulting in a final sample of 172 authors. The response rate was 45% (78 respondents).

### Quantitative analysis

#### Dependent variable

The main dependent variable of this study was the number of QRPs in the reporting of messages and conclusions in HSR publications. QRP’s were identified by RG, JM, and a third assessor. Two reviewers independently assed the HSR publications. Absence and presence of QRP’s was discussed for all publications and identified through mutual consensus. The data were obtained from one of our previous studies, that provides more details on the applied methodology for identifying QRPs [8].

#### Independent variables

The items (i.e., questions) included in the survey questionnaire derived from three major domains as described above (i.e., the individual, institutional, and scientific environment). An exploratory factor analysis was conducted to identify factors underlying items within each domain. The factors identified in the exploratory analyses were named as much as possible in alignment with the factors in our theoretical framework.

We used the factors identified from the factor analysis as independent variables in our analyses. The methods and results of the factor analysis are further described in supplementary material 4.

Considering the explorative nature of our study, no assumptions were made regarding the relative importance of factors within and between domains. Due to our explorative aim, we decided to include all factors resulting from the factor analyses.

#### Other characteristics

We collected several personal characteristics in the survey (i.e., age, working experience as scientific researcher, academic background, academic position, number of publications co-authored, and journal’s impact factor). We described sample characteristics based on these variables.

#### Statistical analysis

The basic characteristics of study samples were described based on the measurement scale of the variables. Categorical variables (nominal / ordinal) were presented as frequency and percentage, whereas numerical variables (interval/ratio) were presented using mean and standard deviation.

We conducted bivariate analysis using simple Poisson regression. Poisson regression was chosen considering the nature of the outcome (number of QRPs) as count data with a relatively small mean value. This analysis specifically assessed the association between each factor score and the number of QRPs in HSR publications. The analysis was also intended to reduce the number of factors which were included in the multivariate model.

Following the bivariate analysis, we applied multiple Poisson regression to further assess the association between the factor domains and the number of QRPs in HSR publications. For the purpose of model development, we provided four models in our multivariate analysis to ensure the stability of our results. The first two models were crude models (unadjusted) and included 12 factors from the bivariate analyses that exhibited a significant association with QRPs (*p*s < .3). The last two models included number of years of work experience as scientific researcher, as well as the journal’s impact factor, to examine the influence of these variables’ influence on the quality of reporting. For easier interpretation, we provided the coefficient of each explanatory variable (B), exponential form of the coefficient (Exp B) and the 95% confidence interval (95% CI). The goodness of fit of all models was checked using the chi-square goodness of fit test as part of the Poisson regression procedure, with results suggesting all models demonstrated good fit.

Considering that the factors we used as independent variables may be interrelated, we checked for collinearity in our regression model. Results from the correlation matrix in the exploratory factor analysis procedure showed 35 of 171 pairs between scales (20%) were significantly but not strongly correlated (*r*s < 0.3). Hence, these findings suggested no multi-collinearity issues in our analysis.

Because first and last authors have different roles in the writing of scientific publications, we provided additional stratified analysis between first and last authors to further explore the nature of the association between these factors and the number QRPs. All analyses were conducted using IBM SPSS version 25.

### Ethics approval

A waiver for ethical approval was obtained for this study from the medical ethics review committee at Amsterdam UMC. To avoid negative consequences for participants, each participant and publication was assigned a unique identification number. Extracted data were entered in SPSS using this number to separate author information from the study data.

## Results

Of the survey participants, 51.3% were a first author and 48.7% were a last author. PhD students (25.6%) and professors (29.5%) were the most frequent academic positions in our sample. First authors were predominantly PhD students (50.0%), whereas the last authors were predominately professors (57.9%). Both first (40%) and last (28.9%) authors primarily had an academic background in the social sciences (40%). Last authors were older, had longer working experience as a scientific researcher, and reported a larger number of publications co-authored as compared to first authors. The journal impact factor of the publications was similar between last and first authors. The number of QRPs per publication was slightly higher for the last authors than first authors. The basic characteristics of the study sample are provided in Table 2. There are 12 publications corresponding to both first and last author, 28 publications correspond only to a first author, and 26 publications correspond only to a last author.

**Table 2:** Basic characteristics of survey respondents

	**Overall**		**First author**		**Last author**	
	**n**	**%**	**n**	**%**	**n**	**%**
**Author status**						
First author	40	51.3	–	–	–	–
Last author	38	48.7	–	–	–	–
**Research position (January 2016)**						
PhD student	20	25.6	20	50.0	0	0.0
Post-doctoral researcher	10	12.8	10	25.0	0	0.0
Senior researcher	13	16.7	5	12.5	8	21.1
Assistant professor	6	7.7	2	5.0	4	10.5
Associate professor	5	6.4	1	2.5	4	10.5
Professor	23	29.5	1	2.5	22	57.9
Other	1	3.1	1	2.5	0	0.0
**Academic background**						
Social sciences	27	34.6	16	40.0	11	28.9
Epidemiology	21	26.9	10	25.0	11	28.9
(Health) economics	4	5.1	2	5.0	2	5.3
Other	26	33.3	12	30.0	14	36.8
	**Mean**	**SD**	**Mean**	**SD**	**Mean**	**SD**
**Age of participant (years)**	45.21	11.67	37.90	10.08	52.92	7.60
**Working experience as scientific researcher (years)**	15.83	9.78	9.44	6.99	22.55	7.63
**Average number of publications co-authored per year**	4.88	1.95	3.43	1.47	6.42	0.98
**Journal Impact Factor of publication**	2.07	1.74	2.08	1.50	2.06	1.99
**Number of QRPs in publication**	6.04	3.46	5.83	3.50	6.26	3.45

### Bivariate analyses

Table 3 depicts findings from bivariate analyses examining the relationship between each factor from the individual, institutional, and scientific environment domain and the number of QRPs. Of the five factors in the individual domain, “pressure to create societal impact” (Exp B = 1.34, 95% CI [1.18, 1.51]) and “self-efficacy” (Exp B = 0.84, 95% CI [0.72, 0.98]) exhibited significant associations with the number of QRPs. For institutional factors, only “specific training in reporting messages and conclusions” (Exp B = 0.85, 95% CI [0.77, 0.93]) exhibited a significant association with the number of QRPs. Stakeholder influence (Exp B = 1.16, 95% CI [1.06, 1.27]) was the only factor from the scientific environmental domain that exhibited a significant association with the number of QRPs.

**Table 3:** Results of bivariate Poisson regression analysis examining individual, institutional, and scientific environment domain factors’ associations with number of QRPs

Domain	Factors	B	SE	*p*-value	Exp(B)	95% CI
**Individual**	Ambition in science	0.075	0.090	0.404	1.08	0.90–1.28
	Self-efficacy	−0.171	0.079	0.031	0.84	0.72–0.98
	Perception of received training	−0.008	0.081	0.917	0.99	0.85–1.16
	Confidence in writing	−0.111	0.083	0.180	0.89	0.76–1.05
	Pressure to create societal impact	0.289	0.064	0.000	1.34	1.18–1.51
	Perception of contribution to science.	0.102	0.069	0.139	1.11	0.97–1.27
**Institution**	Specific training in reporting messages and conclusions	−0.169	0.048	0.000	0.85	0.77–0.93
	Competitiveness	0.089	0.053	0.091	1.09	0.98–1.21
	Data storage	−0.045	0.054	0.404	0.96	0.86–1.06
	Feedback culture at institute	0.022	0.051	0.666	1.02	0.93–1.13
	Social support	−0.034	0.084	0.683	0.97	0.82–1.14
	Media policy	0.018	0.059	0.757	1.02	0.91–1.14
	Influence of funders	−0.068	0.065	0.296	0.93	0.82–1.06
**Environment**	Creating exciting conclusion	0.019	0.070	0.783	1.02	0.89–1.17
	Media contact	−0.018	0.061	0.769	0.98	0.87–1.11
	Pressure from scientific culture	0.048	0.071	0.495	1.05	0.91–1.21
	Suspicions of co-workers	−0.100	0.071	0.160	0.91	0.79–1.04
	Journal practice	−0.089	0.067	0.186	0.92	0.80–1.04
	Stakeholder influence	0.146	0.047	0.002	1.16	1.06–1.27
	Co-author conflict of interest	−0.100	0.057	0.082	0.91	0.81–1.01
	Conflict between co-authors	−0.077	0.045	0.087	0.93	0.85–1.01

### Multivariate analyses

Results of multivariate analyses are presented in Table 4. Of the four models in our analysis, three factors i.e., “pressure to create societal impact”, “specific training”, and “co-author conflict of interest” consistently exhibited a significant association with the number of QRPs.

**Table 4:** Multivariate analysis between factors from individual, institutional, and scientific environment domains with number of QRPs using Poisson regression

	Model 1^**a**^			Model 2^**b**^			Model 3^**a**^			Model 4^**b**^		
	B	Exp(B)	95% CI	B	Exp(B)	95% CI	B	Exp(B)	95% CI	B	Exp(B)	95% CI
Intercept	1.397	4.04	1.24–13.10	1.501	4.48	2.07–9.72	1.633	5.11	1.50–17.45	1.634	5.12	2.27–11.54
Journal impact factor	–	–	–	–	–	–	0.051	0.95	0.89–1.01	−0.053	0.95	0.89–1.01
Working duration	–	–	–	–	–	–	−0.001	0.99	0.98–1.01	−0.000	1.00	0.99–1.01
**Individual**												
Self efficacy	−0.058	0.94	0.78–1.13	−0.130	0.98	0.75–1.03	−0.055	0.95	0.79–1.14	− 0.129	0.88	0.75–1.03
Confidence in writing	−0.029	0.97	0.81–1.17	–	–	–	−0.018	0.95	0.78–1.15	–	–	–
Pressure to create societal impact	0.268	1.31	1.14–1.50	0.226	1.25	1.10–1.42	0.245	1.28	1.11–1.47	0.199	1.22	1.07–1.39
Perception of contribution to science.	0.100	1.11	0.94–1.30	–	–	–	0.084	1.09	0.93–1.28	–	–	–
**Institution**												
Specific training	−0.171	0.84	0.76–0.93	−0.155	0.86	0.78–0.94	−0.165	0.85	0.77–0.94	−0.147	0.86	0.78–0.95
Competitiveness	0.057	1.06	0.95–1.18	–	–	–	0.052	1.05	0.94–1.18	–	–	–
Influence of funders	−0.078	0.93	0.79–1.08	–	–	–	−0.075	0.93	0.80–1.08	–	–	–
**Environment**												
Suspicion of co-workers	−0.002	0.99	0.84–1.18	–	–	–	0.002	1.01	0.85–1.20	–	–	–
Journal practice	−0.027	0.97	0.84–1.13	–	–	–	−0.022	0.98	0.84–1.13	–	–	–
Stakeholder influence	0.050	1.05	0.95–1.17	0.091	1.10	0.99–1.21	0.065	1.07	0.96–1.19	0.103	1.11	1.00–1.22
Co-author conflict of interest	−0.151	0.86	0.76–0.98	–	–	–	− 0.157	0.85	0.75–0.97	–	–	–
Conflict between co-authors	−0.016	0.98	0.89–1.10	–	–	–	−0.012	0.98	0.89–1.09	–	–	–

^a^Included domains with *p*-value < 0.30 in bivariate analysis; ^b^Included domains with *p*-value < 0.05 in bivariate analysis

In the fully-adjusted model (i.e., model 3), a one-point increase on the “pressure to create societal impact” item was associated with a 28% increase in the number QRPs in an HSR publication (Exp B = 1.28, 95% CI [1.11, 1.47]). Conversely, a one-point increase on the “specific training in reporting messages and conclusions” was associated with a 15% decrease in the number QRPs of an HSR publication (Exp B = 0.85, 95% CI [0.7, − 0.94]). A one-point increase on the “co-author conflict of interest” item was associated with a 15% decrease in the number of QRPs in an HSR publication (Exp B = 0.85, 95% CI [0.75, 0.97].

### Stratified analyses between first and last authors

Results from stratified analyses between first and last authors, along with results of multivariate analyses using the fully adjusted model, are included in Table 5. A complete description of our stratified analysis with all applied models can be found in the supplementary material 5.

**Table 5:** Comparison of factors associated with the number of QRPs in reporting of messages and conclusions in HSR publication between first and last authors

Factors^**a**^	First author			Last author		
	B	Exp(B)	95% CI	B	Exp(B)	95% CI
Intercept	0.248	1.28	0.18–0.93	2.212	9.33	1.47–56.60
Journal impact factor	−0.114	0.89	0.80–1.01	− 0.066	0.94	0.86–1.02
Working duration	0.004	1.01	0.98–1.03	0.005	1.01	0.98–1.02
**Individual**						
Ambition in science	0.306	1.36	0.99–1.85	–	–	–
Self-efficacy	–	–	–	−0.198	0.82	0.59–1.14
Perception of received training	−0.139	0.87	0.68–1.14	0.253	1.29	0.94–1.75
Confidence in writing	–	–	–	−0.360	0.70	0.48–1.01
Pressure to create societal impact.	0.216	1.24	1.02–1.51	0.175	1.19	0.90–1.58
Perception of contribution to science.	0.192	1.21	0.94–1.55	–	–	–
**Institution**						
Specific training	−0.170	0.84	0.72–0.98	− 0.058	0.94	0.77–1.15
Competitiveness	–	–	–	0.099	1.10	0.88–1.39
Data storage	−0.112	0.89	0.73–1.10	–	–	–
Feedback culture at institute	0.218	1.24	1.05–1.47	−0.094	0.91	0.75–1.10
Influence of funders	−0.173	0.84	0.70–1.02	–	–	–
**Environment**						
Creating exciting conclusion	0.060	1.06	0.79–1.44	−0.052	0.95	0.73–1.24
Suspicions of co-workers	–	–	–	0.047	1.05	0.76–1.44
Journal practice	−0.075	0.93	0.77–1.12	–	–	–
Stakeholder influence	0.052	1.05	0.90–1.24	−0.012	0.98	0.77–1.27
Co-author conflict of interest	–	–	–	−0.083	0.92	0.75–1.12
Conflict between co-authors	–	–	–	−0.118	0.89	0.74–1.06

^a^The multivariate models included different factors (independent variables) between first author and last author analysis resulting the bivariate analysis. For a complete description please refer to the supplementary material 5

For first authors, findings indicated “specific training” reporting messages and conclusions (Exp B = 0.84, 95% CI [0.72, 0.98]) was associated with fewer QRPs. “Feedback culture” at their research institute (Exp B = 1.24, 95% CI [1.05, 1.47]) and “pressure to create societal impact” (Exp B = 1.24, 95% CI [1.02, 1.51]) contribute to a higher number of QRPs. For last authors no significant relationship was identified between factors and QRPs.

## Discussion

The aim of this study was to explore the possible association between individual, institutional, and scientific environment factors and inadequacies in the reporting of messages and conclusions in scientific HSR publications.

We identified three factors independently associated with QRPs in the reporting of messages and conclusions in scientific HSR publications i.e., “pressure to create societal impact”, “specific training reporting messages and conclusions” and “co-author conflict of interest”. Stratification between first and last author indicated different factors related to the occurrence of QRPs.

### Interpretation

Our results indicated three factors are independently associated with QRPs in the reporting of messages and conclusions in HSR literature. The other factors in the assessed framework, however, are not irrelevant. All included factors may relate to multiple aspects of the publication process and are worth addressing in future studies on QRPs. Our study was explorative, and we therefore recommend further empirical research on the resulting factors.

The association between a higher number of QRPs and the factor “pressure to create societal impact” facilitates important insights on the current research culture of HSR. HSR is often intended for practical intervention [23]. To improve the connection between HSR and policy and practice across the entire field of HSR, researchers are stimulated to spread their findings via societal publications to policy makers, professionals and the public [24]. HSR researchers may anticipate their societal impact when writing their scientific publications. Hence, they may be likely to unconsciously adapt their language and writing to present concrete and actionable conclusions suited to attract the attention of the media or the professional community [25]. It is generally assumed that pressure to create societal impact pushes authors to overstate conclusions in press releases or other societal publications. However, the current findings suggest a possible effect on scientific reporting as well. Currently, researchers may not have the means to responsibly create societal impact or have difficulty aligning their scientific messages with societal messages. Not all researchers are equally equipped for this task due to differences in research experience or practical experience in healthcare. While research and practice in HSR are traditionally intertwined, researchers from other disciplines, like biomedical research, will similarly experience an increasing need to create societal impact. With the increasing attention societal impact by the scientific community at large, future studies addressing scientific reporting should take into account the association between the perception of research impact and reporting in scientific publications.

“Specific training in reporting messages and conclusions” was associated with a lower number of QRPs. The positive association between training and the improvement of reporting skills has been identified in previous studies [26, 27]. The practice of phrasing and reporting is an inherent part of extracting messages and conclusion from results. From our focus groups we know that some researchers receive training that specifically combine these skills. Because the participants self-reported on their level of specific training, our findings highlight that some courses offered by HSR institutions in the Netherlands may provide researchers with helpful tools to improve their writing. Moreover, researchers may be capable in recognizing they need more specific training. Institutions should assure that those who need specific training in reporting messages and conclusions will be able to obtain it.

“Co-author conflict of interest” was associated with a lower number of reporting adequacies. This finding contradicts the assumption that research quality generally decreases when a conflict of interest arises. A possible explanation may be that awareness of a conflict of interest by co-authors may have stimulated a more nuanced or careful interpretation of the research findings. Policies in place at HSR institutions could assure that those conflicts of interests are positively mitigated and result in more attention to research conduct [28].

One method used by research institutions to force a stimulating debate is to introduce structured peer-feedback [29]. Although some institutions in the Netherlands have invested in structuring feedback support for their researchers, feedback culture was not associated with a lower number of QRPs in the current study. Surprisingly, the analyses differentiating between first and last authors indicated that feedback culture may contribute to more QRPs for first authors. Feedback structures differ for each institution, and some might not be aimed sufficiently at the interpretation and reporting of messages and conclusions. It could thus be worthwhile to investigate how feedback structures can better support authors and what type of feedback culture would specifically create a stimulating debate. The assessment form developed for assessing QRPs in scientific publications might guide structured feedback on reported messages and conclusions specifically, and may be retrieved from the supplementary material 1 in our previous open access publication [6].

Our analyses further indicate that factors contributing to QRPs may be different for first and last authors. First authors may contribute to more QRPs when they experience more pressure to create societal impact and a positive feedback culture. They may contribute to fewer QRPs when they receive more specific training in reporting messages and conclusions. Additional research on the unique roles of first and last authors in the prevention of QRPs when reporting messages and conclusions in scientific HSR publications is recommended.

### Limitations

The main strength of our approach was our mixed methods design. By constructing a framework from the experiences of a sample of health services researchers, we could tailor the survey to our study participants. Moreover, most research on research integrity is derived from self-report. Our assessment of QRPs provides a more impartial approach.

Considering the large turnover of research staff and PhD students at each institution, a response of 45% may be optimal. The average number of QRPs is similar to that of all assessed HSR publications. Nevertheless, non-respondents might have rejected participation because of time pressures or a lack of communication with the HSR community. The relatively small sample size in the current study also presents as a limitation and necessitates replication in larger and more diverse samples. Further, due to our explorative aim, we decided to include factors with a lower threshold of reliability. In follow-up research we recommend to improve the factors’ reliability.

We acknowledge the publications we analysed are nested in the thirteen participating Dutch institutions, which may influence the associations between institutional factors and QRPs. However, intraclass variation cannot be fully avoided. Institutions differ in structural conditions/resources, social conditions, role of supervisors, and cultural conditions. Consequently, associations may be attributed to an institutional factor although it is dependent on the context of a particular institution. A multilevel analysis including the institution in which the publication is nested would be the ideal option to address this issue. However, such an analysis would likely have required a larger dataset to provide a robust estimation of effects, particularly given the fact that publications are typically written by authors from multiple institutions. Considering the relatively small sample size and the explorative nature of our study, a single-level regression was a more appropriate choice. Further study with a larger data set and clearly distinguished institutions will allow for a more sophisticated analysis technique to confirm findings from our study.

The assessed publications were published in 2016. Researchers might not have had a very vivid memory of their working experience 2 years prior. This would have made our connections between publication and person somewhat less reliable. Nevertheless, we do not expect institutional or scientific factors to have changed significantly in the course of 2 years. Risk for recall bias was likely minimal.

The researchers and studies included in this study all originated from Dutch research institutions. Institutional structures and individual experiences of research culture will be different across countries. For instance, Dutch universities employ PhD students, who are often the primary authors of research papers, rather than provide scholarships. Moreover, they share supervisory structures requiring at least two supervising seniors. Nevertheless, HSR researchers and institutions often deal with similar challenges as those encountered in the Netherlands, including publication pressures and creating societal impact. Aspects of the results from the current study are thus likely to provide a helpful guide for HSR institutions internationally.

### Implications and recommendations for policy and practice

In the field of HSR there is potential for a different set of guidelines for assessing QRPs as compared to the biomedical field. Due to the different research questions and methodologies applied, the research studies presented in HSR should adhere to the standards set by reporting guidelines and tools designed specifically for the field [30]. Peer reviewers selected for these studies should therefore be familiar with the standards of the field. Prior to publication, QRPs in the reporting of messages and conclusions may be intercepted during peer review and brought to the attention of the authors. We thus encourage journals to request peer-reviewers to assess a publication for the presence of the discussed QRPs.

Our study moreover identified factors that are best addressed through changes by research institutions. Results should stimulate awareness within the HSR community internationally. In support of a more responsible translation of findings to policy and practice, they should address the identified factors to contribute to better reporting in scientific HSR publications. We recommend the development of institutional interventions to encourage responsible reporting of messages and conclusions in HSR. Specialized writing courses and workshops may increase writing confidence [27]. Specific training already in place at research institutions on writing discussions and conclusions should be extended to all health services researchers who do not have access. HSR institutions should further prioritize providing a positive feedback culture by stimulating debate and making conflicting interests explicit. They should moreover, introduce systematic changes such as organizing peer-review with engaging discussions, and providing sufficient time and support in balancing scientific reporting and creating societal impact. Across the HSR field, institutions are already taking actions to assure responsible research practices, thus we recommend them to strengthen the coherence of their efforts, also by collaboration.

## Conclusion

Experienced pressure to create societal impact is associated with a higher number of QRPs in the reporting of messages and conclusions in HSR publications. Specific training in reporting messages and conclusions, and awareness of co-author conflict of interests are related to fewer QRPs in HSR publications. This study was exploratory and we therefore recommend further research on the identified factors. Our results should stimulate awareness within the field of HSR internationally on opportunities better support reporting in scientific HSR publications, and thus a more responsible translation of findings to policy and practice.

## Supplementary information

**Additional file 1.**

**Additional file 2.**

**Additional file 3.**

**Additional file 4.**

**Additional file 5.**

## Data Availability

The datasets used and/or analysed during the current study are available from the corresponding author on reasonable request. Project files for are available in the Figshare public repository: 10.21942/uva.9255335.

## Abbreviations

CI: Confidence Interval
B: Beta
Exp(B): Exponentiation of the Beta coefficient
HSR: Health Services Research
QRP: Questionable Research Practices
SD: Standard Deviation
SE: Standard Error
ZonMw: The Netherlands Organization for Health Research and Development
