# Supplementary material for: Individual, institutional, and scientific environment factors associated with questionable research practices in the reporting of messages and conclusions in scientific health services research publications
Source: BMC Health Serv Res. 2020 Sep 3;20:828. doi: 10.1186/s12913-020-05624-5 (PMC7469341; doi:10.1186/s12913-020-05624-5)
Supplement: Supplementary file 1 — Additional file 1. [file 12913_2020_5624_MOESM1_ESM.docx]

**Focus group guide april/mei 2018**

**[English translation from original language, Dutch]**

**Introduction**

Introduction facilitators and participants

Information project (with PowerPoint presentation)

1. Explained goal project, origin project

**Goal focus group**

During this focusgroep the following subjects are discussed

1. Your experience with reporting of conclusions and messages in HSR
2. How you are supported in responsibly reporting HSR

**Privacy protection**

The focusgroep will take 1.5 hours. With your consent, the conversation will be recorded, and the spoken data will be transcribed. After analyses a summary will be shared with the participants for verification. The focus group will be confidential. Only researchers working on this project will have access to the content of the conversation. The reporting of the findings will take place on an aggregated level, to avoid traceability to a person or institution.

First, we will shortly explain our definition of ‘questionable reporting of conclusions and messages’.

*To report, either intentionally or unintentionally, conclusions or messages that may lead to incorrect inferences and do not accurately reflect the objectives, the methodology or the results of the study.*

Facilitator: this definition is not part of the discussion. Show examples of possible QRPs (PowerPoint)

**Question guide**

**Questions regarding problem identification**

1. Do you recognize tensions in the reporting of conclusions, messages or recommendations?
   1. Do you think this is a problem? If yes, why?

**Experiences with reporting conclusions, messages and recommendations**

1. Could you tell us about your own experiences regarding responsible reporting of conclusions and messages?
   1. How do you go about reporting different parts of a publication? i.e. abstract, goal and research question, context of evidence, limitations, conclusions based on results and recommendations?
   2. What helps you with reporting of these sections?
2. What are possible difficulties in responsibly reporting conclusions messages or recommendations?
   1. Could you explain why you think these sections are difficult to report? i.e. abstract, goal and research question, context of evidence, limitations, conclusions based on results and recommendations?
3. How does the institute where you work pay attention to reporting?
   1. Does the institute where you work have any policy that stimulates responsible reporting?
      1. If yes, what policy?
   2. Does this policy help with reporting?
      1. Why yes/no?
4. Are there practices that your institute could implement to stimulate responsible reporting?
   1. If yes, what practices?
   2. How could these practices help you?
5. How do you think the scientific system supports you in responsible reporting conclusions and messages?
   1. Why do these practices (not) support you?
   2. Who is responsible for addressing these actions, in your opinion?

**Closing**

1. Do you have any other suggestions that could contribute to responsible reporting of messages and conclusions?

Thank you for your participation and mention privacy protection.

A report will be sent for validation.
