# Supplementary material for: Individual, institutional, and scientific environment factors associated with questionable research practices in the reporting of messages and conclusions in scientific health services research publications
Source: BMC Health Serv Res. 2020 Sep 3;20:828. doi: 10.1186/s12913-020-05624-5 (PMC7469341; doi:10.1186/s12913-020-05624-5)
Supplement: Supplementary file 2 — Additional file 2. [file 12913_2020_5624_MOESM2_ESM.docx]

**Supplementary material 2**

**Additional information to the qualitative methods on the development of a framework of factors contributing to reporting inadequacies in the reporting of messages and conclusions in Health Services Research**

This document describes the qualitative methods on the development of a framework of factors contributing to reporting inadequacies in messages and conclusions in Health Services Research (HSR).

Factors potentially associated with reporting inadequacies in HSR were identified through an explorative review of scientific literature, 14 semi-structured interviews with leaders/representatives (n=19) of 13 participating HSR institutions and 13 focus groups with junior/PhD researchers (n=57) at the participating HSR institutions.

**Explorative literature review**

First, an explorative literature review was conducted searching for factors related to the conduct of questionable research practices in general. Initial search terms included in different order and combination: ‘questionable research practices’, ‘factors’, ‘questionable conclusions’, ‘misconduct’ and ‘spin’. The majority of publications were included through snowball sampling. Publications were included if they mentioned possible factors that might impact the conduct of questionable research practices, or provided definitions of the above key terms.

After identifying a body of literature, we screened all publications for factors possibly related to questionable research practices, and in particular might relate to the reporting of messages and conclusions. A broad selection of factors was included in an initial framework. This framework was used as a basis for semi-structured interviews with the leaders and representatives of the thirteen participating institutions. The publications identified are listed on page 4-6 of this supplement.

**Semi-structured interviews**

Second, nineteen leaders/representatives of the thirteen participating HSR institutions were interviewed during fourteen semi-structured interviews. Two interviews took place at one institution as it was represented by two departments. Three interviews were conducted with both the institute leader and a second representative. One of the interviews included three representatives of an institution. The aim of the interviews was to discuss our draft of Questionable Research Practices (QRPs), referred to in this paper as ‘reporting inadequacies’, and identify additional measurable reporting inadequacies in the reporting of messages and conclusions in HSR, explore potential causes of reporting inadequacies in messages and conclusions, and to discuss experiences of the institute leaders with these reporting inadequacies. A semi-structured interview guide was developed by the project team, that is published as an attachment to Gerrits et al. (2019) in BMJ open^[[1]](#footnote-1)^. We presented the interviewees with the preliminary framework of factors identified in the literature. The draft framework was iteratively adjusted, i.e. after each interview we drafted a new version including the findings of the previous interviews, which we then presented during each following interview.

Interviewees were approached through e-mail to schedule an appointment. Two researchers conducted the interviews of which thirteen took place at the participating institutions and one interview took place in a public space. During the first interview, both interviewers were present to align their interviewing approach. The remainder of the interviews were equally divided between the interviewers. The interviews lasted one hour. In concordance with ethical guidelines, the goal of the interview was explained at the start of the interview and permission to audio-record the interview was obtained.

With the support of the recordings, a report was written and shared with the interviewees for validation. From these validated reports and the updated list of reporting inadequacies, a final list of reporting inadequacies was drafted.

**Focus groups**

Third, we applied a focus group approach to explore possible factors related to the occurrence of reporting inadequacies in HSR.

**Recruitment of focus group participants**

The thirteen participating institutions were asked to support the organization of these focus groups. One institution declined as they were engaged in education during the research period. The intended duration of the focus groups was one and a half hour. The intended size of the focus groups was six to ten. Focus group participants were recruited through a contact person at the respective institutions. These contact persons were provided with a full description of the aims and methods of the focus groups. When potential participants were identified, a date for the focus group was determined. We emphasized that participation was completely voluntary, and no requirement by the participating institutions.

In total,13 focus groups at 12 institutions (in one institute two departments participated) were conducted, including 57 participants. The average number of participants was 4-5.

**Setting**

All focus group discussions took place at the institute locations to ease traveling expenses and time of the participants. Focus groups were conducted between April 2018 and October 2018.

During 11 focus group two researchers were present, one acting as facilitator and one as note taker. The facilitator ensured that the session progressed fluently, while the note taker took notes of the content and made sure that all the topics are covered. During the two last focus groups, one researcher was present, who both facilitated and took notes of the conversation. Participants were fully informed on the goal of the study. They were informed through e-mail. At the start of each focus group, the goal of the study was explained and it was clearly expressed participation was voluntary. Consent to participate by all participants was confirmed at the start of each focus group.

With permission of the focus group participants all focus groups were audiotaped. Ten recordings were transcribed ad verbatim. For all thirteen focus groups, a report was written. All participants were sent their respective reports for validation by e-mail.

The focus groups were guided by a focus group guide addressing the experience of researchers on factors that might influence reporting inadequacies, experienced good practices and discovering new good practices in the field of HSR. The guide is provided in supplementary material 5. The transcriptions were analyzed through content analyses. The results from this analysis was confirmed with the reports of the remaining three focus groups.

**Analyses**

The transcripts were analyzed by inductive coding. The following steps were used to systematically analyze the transcribed data; (1) raw data files were prepared in the analyses program MaxQDA, (2) the full transcriptions were read closely, (3) themes were created, (4) overlapping codes and themes were connected (5) the themes were iteratively refined. Throughout the whole coding process, emerging themes were regularly compared and discussed by the two researchers and the research group.

First, JW and RG independently analyzed two transcripts. They then compared their codes and agreed upon an initial coding scheme. JW then continued with the analyses of the eight remaining transcripts. RG regularly checked the coding performed by JW. After the analyses of 10 focus group transcripts, saturation was reached. RG validated the results from the analyses with the focus group reports from the last three focus groups.

Factors resulting from the focus group analyses were added to the framework resulting from the semi-structured interviews with leaders and representatives of the institutions.

**Ethics**

A waiver for ethical approval was obtained from the METC at the Academic Medical Center.

**Framework**

Identified factors were included in a theoretical framework existing of three domains: the individual, institutional, and scientific environment domain. The individual domain concerns characteristics that are bound to the individual researcher. These may concern characteristics such as research experience and self-efficacy. The institutional domain includes factors that are controlled by the institution that houses the researcher. These include institutional culture, facilities, interactions and policies that may affect the writing and publication experience of the researcher. For example, an institution may have an (unofficial) policy to stimulate a certain number of publications per year. The scientific environmental domain includes those factors that take place outside of the control of the institution, and belong to the scientific culture or system a researcher is part of. These are the factors that institutions have no direct control over. For example, factors are scientific journal policy including word length or use of reporting checklists, or collaboration with researchers and stakeholders outside the researching institution. Factors within each domain may be influenced by factors in other domains.

**Framework and included factors**

| **1**  **Research environment ‘culture’** | **2**  **Institutional conditions** | **3**  **Individual researcher characteristics** |
| --- | --- | --- |
| **Funding**   - Funding rewards innovation & novelty - Demands of the funder   **Valorisation of research outcomes**   - Revenue model - Public media - Media pressure   **Policies & practices scientific society**   - Competition for research positions - Journal policies & practices - Peer review process - Pressure to publish ‘exciting’ articles   **Collaborating partners**   - Conflicts of interest   **Research beneficiaries / stakeholders**   - Usefulness: study designed without proper consideration of the value for e.g. patients | **Structural conditions/resources**   - Education - Reward system / incentives - Presence and adherence to a Research code - Recruitment & selection researchers - Presence of formal quality policy - Transparency study materials/data   **Social conditions**   - Opportunities for peer-discussion - Presence of colloquia for article discussion - Review of pre-publication findings - Competitiveness   **Role of supervisors**   - Task perception - Workload - Social skills (in supervision, and collaboration)   **Cultural conditions**   - Ideology institute - Functioning of the ICT infrastructure - Open organisation culture | **Motivation**   - Promotion - Respect from peers - Focus on short-term success - Ideology   **Capabilities**   - Research training in HSR - Writing skills - Training research integrity - Social skills - Self-efficacy (to stand up to pressure)   **Working conditions**   - Workload - Work pressure   **Perceptions**   - Self-perception - Perception of others   **Personality traits**   - Narcissism |

**Publications identified in the exploratory review**

Asman, O., Melnikov, S., Barnoy, S., & Tabak, N. (2017). Experiences , behaviors , and perceptions of registered nurses regarding research ethics and misconduct. *SAGE*, 1–11.

Bohannon, J. (2013) Who's Afraid of Peer Review? Science magazine 342 (6154): 60-65.

Bosch, X. (2014). Improving biomedical journals ’ ethical policies: the case of research misconduct. *Journal of Medical Ethics*, *40*, 644–646. https://doi.org/10.1136/medethics-2013-10182

Bouter, L. M. (2015). Commentary: Perverse incentives or rotten apples?. *Accountability in research*, *22*(3), 148-161.

Braakhekke, M., Scholten, I., Mol, F., Limpens, J., Mol, B. W., & Veen, F. Van Der. (2017). Selective outcome reporting and sponsorship in randomized controlled trials in IVF and ICSI. *Human Reproduction*, *32*(10), 2117–2122. <https://doi.org/10.1093/humrep/dex273>

Chalmers, I., & Glasziou, P. (2009). Avoidable waste in the production and reporting of research evidence. *Obstetrics & Gynecology*, *114*(6), 1341-1345.

Crain, A. L., Martinson, B. C., & Thrush, C. R. (2013). Relationships between the Survey of Organizational Research Climate (SORC) and self-reported research practices. *Science and engineering ethics*, *19*(3), 835-850.

English, T., Antes, A. L., Baldwin, K. A., & Dubois, J. M. (2017). *Development and Preliminary Validation of a New Measure of Values in Scientific Work*. *Science and Engineering Ethics*. Springer Netherlands. https://doi.org/10.1007/s11948-017-9896-0

Fanelli, D., Costas, R., & Ioannidis, J. P. A. (2017). Meta-assessment of bias in science. *PNAS*, *114*(14). https://doi.org/10.1073/pnas.1618569114

Glasziou, P., Altman, D. G., Bossuyt, P., Boutron, I., Clarke, M., Julious, S., ... & Wager, E. (2014). Reducing waste from incomplete or unusable reports of biomedical research. *The Lancet*, *383*(9913), 267-276.

Godecharle, S., Nemery, B., & Dierickx, K. (2014). Heterogeneity in European research integrity guidance: relying on values or norms?. *Journal of Empirical Research on Human Research Ethics*, *9*(3), 79-90.

Haddaway, N. R., Woodcock, P., Macura, B., & Collins, A. (2015). Making literature reviews more reliable through application of lessons from systematic reviews. *Conservation Biology*, *29*(6), 1596–1605. JOUR. https://doi.org/10.1111/cobi.12541

Horrobin, D. F. (1999). Beyond conflict of interest. Non-financial conflicts of interest are more serious than financial conflicts. *The BMJ*, *318*(7181), 466-466.

Horton, R. (1995). The rhetoric of research. *BMJ: British Medical Journal*, *310*(6985), 985.

Ioannidis, J. P., & Khoury, M. J. (2014). Assessing value in biomedical research: the PQRST of appraisal and reward. *Jama*, *312*(5), 483-484.

Ioannidis, J., Tatsioni, A., & Karassa, F. B. (2010). Who is afraid of reviewers’ comments? Or, why anything can be published and anything can be cited. *European journal of clinical investigation*, *40*(4), 285-287.

Jefferson, T., Di Pietrantonj, C., Debalini, M. G., Rivetti, A., & Demicheli, V. (2009). Relation of study quality, concordance, take home message, funding, and impact in studies of influenza vaccines: systematic review. *BMJ*, *338*, b354.

Kleinert, S., & Horton, R. (2014). How should medical science change?. *The Lancet*, *383*(9913), 197-198.

Macleod, M. R., Michie, S., Roberts, I., Dirnagl, U., Chalmers, I., Ioannidis, J. P., ... & Glasziou, P. (2014). Biomedical research: increasing value, reducing waste. *The Lancet*, *383*(9912), 101-104.

Marco, C. A., & Larkin, G. L. (2000). Research ethics: ethical issues of data reporting and the quest for authenticity. *Academic Emergency Medicine*, *7*(6), 691-694.

Martin, B. R. (2013). Whither research integrity ? Plagiarism , self-plagiarism and coercive citation in an age of research assessment. *Elsevier*, *42*(5), 1005–1014. https://doi.org/10.1016/j.respol.2013.03.011

Martinson, B. C., Thrush, C. R., & Crain, A. L. (2013). Development and validation of the survey of organizational research climate (SORC). *Science and engineering ethics*, *19*(3), 813-834.

Marusic, A., Wager, E., Utrobicic, A., Rothstein, H. R., & Sambunjak, D. (2016). Interventions to prevent misconduct and promote integrity in research and publication. *Cochrane Databasae of Systematic Reviews*, (4). https://doi.org/10.1002/14651858.MR000038.pub2

Mazar, N., & Ariely, D. (2015). Dishonesty in scientific research. *The Journal of clinical investigation*, *125*(11), 3993-3996.

Mondragón Barrios, L., Jiménez Tapia, J. A., Meza Mercado, D. M., & Sosa Mora, L. (2017). Regulation and self-regulation of ethical practices in scientific publication. *SALUD MENTAL*, *40*(5). JOUR. https://doi.org/10.17711/SM.0185-3325.2017.029

Olesen, A. P., Amin, L., & Mahadi, Z. (2017). Malaysian researchers talk about the influence of culture on research misconduct in higher learning institutions. *Accountability in Research*, *24*(8), 469–482. https://doi.org/10.1080/08989621.2017.1399358

Oliveira, G. S. De, Jung, M. J., & Mccarthy, R. J. (2015). Discrepancies Between Randomized Controlled Trial Registry Entries and Content of Corresponding Manuscripts Reported in Anesthesiology Journals. *Ovid Insights*, *121*(4), 1030–1033. https://doi.org/10.1213/ANE.0000000000000824

Sharma, O. P. (2015). Ethics in Science. *Indian Journal of Microbiology*, *55*(3), 341–344. https://doi.org/10.1007/s12088-015-0532-x

Smaldino, P. E., & McElreath, R. (2016). The natural selection of bad science. *Royal Society Open Science*, *3*(9), 160384.

Steen, R. G., Casadevall, A., & Fang, F. C. (2013). Why Has the Number of Scientific Retractions Increased ? *PLoS ONE*, *8*(7), 1–9. https://doi.org/10.1371/journal.pone.0068397

Suhrbier, A., & Poland, G. A. (2013). Are Impact Factors corrupting truth and utility in biomedical research? *Elsevier*, *Vaccine 31*, 6041–6042.

I Tijdink, J. K., Schipper, K., Bouter, L. M., Pont, P. M., De Jonge, J., & Smulders, Y. M. (2016). How do scientists perceive the current publication culture? A qualitative focus group interview study among Dutch biomedical researchers. *BMJ open*, *6*(2), e008681.

II Tijdink, J. K., Bouter, L. M., Veldkamp, C. L. S., Ven, P. M. Van De, Wicherts, M., & Smulders, Y. M. (2016). Personality Traits Are Associated with Research Misbehavior in Dutch Scientists: A Cross-Sectional Study. *PLoS One*, 1–12. https://doi.org/10.1371/journal.pone.0163251

Tijdink, J. K. (2017). Publish & Perish; research on research and researchers. *Tijdschrift voor psychiatrie*, *59*(7), 406–413. English Abstract, Journal Article.

Yavchitz, A., Boutron, I., Bafeta, A., Marroun, I., Charles, P., Mantz, J., & Ravaud, P. (2012). Misrepresentation of randomized controlled trials in press releases and news coverage: a cohort study. *PLoS medicine*, *9*(9), e1001308.

Wang, J., Ku, J. C., Alotaibi, N. M., & Rutka, J. T. (2017). Literature Review Retraction of Neurosurgical Publications: A Systematic Review. *World Neurosurgery*, *103*, 809–814.e1. https://doi.org/10.1016/j.wneu.2017.04.014

Zee, S. Van Der, Anderson, R., Poppe, R., & Jordan, J. (2016). When Lying Feels the Right Thing to Do. *Frontiers in Psychology*, *7*(June), 1–13.

1. Gerrits RG, Jansen T, Mulyanto J, van den Berg MJ, Klazinga NS, Kringos DS. Occurrence and nature of questionable research practices in the reporting of messages and conclusions in international scientific Health Services Research publications: a structured assessment of publications authored by researchers in the Netherlands. BMJ Open. 2019;9(5):e027903. [↑](#footnote-ref-1)
