# Supplementary material for: Individual, institutional, and scientific environment factors associated with questionable research practices in the reporting of messages and conclusions in scientific health services research publications
Source: BMC Health Serv Res. 2020 Sep 3;20:828. doi: 10.1186/s12913-020-05624-5 (PMC7469341; doi:10.1186/s12913-020-05624-5)
Supplement: Supplementary file 3 — Additional file 3. [file 12913_2020_5624_MOESM3_ESM.docx]

**Supplementary material 3**

**Survey on publication practices in health services research**

| **Attitude towards publication practices** | **On the following page different statements regarding publication practices are presented. Please provide your opinion on the following statements.   This section contains one page with statements.** | **Answer option likert scale 1 = Strongly disagree 2 = Disagree 3 = Neither agree nor disagree 4 = Agree 5 = Strongly agree** |
| --- | --- | --- |
| 1 | Please note that the following statements concern peer-reviewed Health Services Research specifically. |  |
| 2 | A catchy title is necessary to get a manuscript published |  |
| 3 | An abstract should be exciting to attract attention |  |
| 4 | It is acceptable to describe conclusions beyond the research questions as long as you stay close to your results. |  |
| 5 | It is acceptable if conclusions deviate slightly from the results as long as that is transparent. |  |
| 6 | Recommendations for policy or practice should always be included in an international peer-reviewed publication. |  |
| 7 | Generalizing findings outside the original context is acceptable to get a manuscript published. |  |
| 8 | It is necessary to compare every single result to supporting and contradictory literature in the discussion section. |  |
| 9 | In the limitations section, it is necessary to discuss potential impact of limitations on the findings. |  |
| 10 | It is acceptable to make concluding statements on causality, even when a study design does not allow for inferences on causation. |  |
| 11 | It is acceptable to use superlatives including 'striking', 'impressive', and 'incredibly', in the discussion section |  |
| 12 | It is acceptable to describe conclusions more powerfully or stronger in public communication about the research (e.g. press releases, news items, LinkedIn messages) than in peer-reviewed publications. |  |
|  | Do you have any additional comments regarding the above statements? | [open answer] |
|  |  |  |
| **Individual Researcher level** | **The following section is about your individual researcher characteristics.   Please indicate your agreement with the following statements.   This section contains one page with statements.** |  |
| 1 | In January 2016, I was employed as project leader or principal investigator. |  |
| 2 | Please indicate your agreement with the following statements. |  |
| 3 | I want to advance my career in science. |  |
| 4 | To advance my career, it is important to produce many scientific publications in international peer-reviewed journals. |  |
| 5 | To advance my career, it is important to have societal impact through e.g. television interviews, media exposure. |  |
| 6 | It is important that my co-workers have a high opinion of my work. |  |
| 7 | When my last manuscript was accepted by a peer-reviewed journal I was extremely happy. |  |
| 8 | Before I start a research project, I have a clear idea of what the results will be. |  |
| 9 | I hope to be surprised by my results. |  |
| 10 | I am disappointed if my results are statistically not significant. |  |
| 11 | I am sufficiently trained in the research methods I apply. |  |
| 12 | I have received sufficient training in writing scientific publications. |  |
| 13 | I find it difficult to write conclusions based on my research findings for peer-reviewed publications. |  |
| 14 | I find it difficult to write recommendations for policy or practice in peer-reviewed publications. |  |
| 15 | I have received sufficient training in research integrity. |  |
| 16 | I have difficulty communicating with my co-authors about the contents of a manuscript. |  |
| 17 | I get nervous when I receive feedback about my manuscript from my co-authors. |  |
| 18 | Generally, when I receive a peer-review from a journal, I accept suggestions even when I do not entirely agree. |  |
| 19 | Most of my peer-reviewed publications are excellently written. |  |
| 20 | My peer-reviewed publications make an important contribution to my scientific field. |  |
|  | Do you have any additional comments regarding the above statements? | [open answer] |
|  |  |  |
| 23 Junior | Generally, when writing a manuscript, I accept suggestions from my supervisors/PI/project leader even if I don't entirely agree. |  |
|  |  |  |
| 23 Senior | Generally, when supervising the writing of a manuscript, I make the final decisions on what text to include in the manuscript. |  |
|  |  |  |
| **Institutional level** | **The following section is about the institute where you worked in January 2016. Please indicate your agreement with the following statements keeping the institute where you worked in January 2016 in mind. This section contains three pages with statements.** |  |
|  |  |  |
| 1 | The institute where I worked provided training (opportunities) regarding the writing of discussion and conclusion sections specifically. |  |
| 2 | The institute where I worked provided sufficient training on presenting in writing or verbally my findings in lay terms. |  |
| 3 | The institute where I worked rewards high quality publications. |  |
| 4 | The institute where I worked rewards high numbers of publications. |  |
| 5 | The institute where I worked stimulates high quality publications as opposed to a high quantity of publications. |  |
| 6 | The institute where I worked kept track of my societal impact. |  |
| 7 | I was aware of the research code of conduct at the institute where I worked and understand its contents. |  |
| 8 | Before I was hired at the institute where I worked, I was asked to demonstrate my writing ability. |  |
| 9 | I received training on the formal quality assurance policy regarding the conduct of research at the institute where I worked. |  |
| 10 | Do you have any additional comments regarding the above statements? |  |
| 11 | I received feedback on my manuscript from my co-workers (not co-authors). |  |
| 12 | I find it helpful to receive feedback on my manuscript from my co-workers. |  |
| 13 | It was mandatory to discuss manuscripts during formal peer-review groups at the institute where I worked. |  |
| 14 | In formal peer-review groups, suggestions were made for the revision in the discussion and conclusion sections of manuscripts. |  |
| 15 | At the institute where I worked, I always discussed my manuscripts in a voluntary peer-review group. |  |
| 16 | It was compulsory to address the review comments received in the peer-review group. |  |
| 17 | There is a strong culture of competitiveness within the department/institute where I worked. |  |
| 18 | I prefered not to share my findings with my co-workers before they were published. |  |
| 19 | At the institute where I worked, ideas for new research studies (proposals) are discussed amongst collegues. |  |
| 20 | At the institute where I worked, I regularly attended a journal club together with my co-workers. |  |
| 21 | At the institute where I worked, I regularly consulted a native English speaker on the writing of my manuscripts. |  |
| 22 | At the institute where I worked, I could easily approach a statistician for help with the interpretation of my findings. |  |
| 23 | When writing a public communication (e.g. press release, executive summary), I was always assisted by a communication expert. |  |
|  |  |  |
| 24 junior | My supervisor/PI/project leader at the institute where I worked did not have enough time to look at my manuscripts. |  |
| 25 Junior | My supervisor/PI/project leader at the institute where I worked has strong social skills. |  |
| 26 Junior | At the institute where I worked, I followed a course in academic writing before January 2016. |  |
|  |  |  |
| 24 Senior | I do not have sufficient time review manuscripts my juniors/PhD students write. |  |
| 25 Senior | I have strong social skills. |  |
| 26 | Who bears final responsibility over the final version of a manuscript and its contents? | 1 = First author / 2 = Last author / 3 = All authors / 4 = The institute - department |
|  | Do you have any additional comments regarding the above statements? | [open answer] |
|  |  |  |
| 27 | The institute where I worked stimulates data sharing and open access policies. |  |
| 28 | The institute where I worked has strict rules on the storage of data and filing of research material. |  |
| 29 | It is common for the institute where I worked to write a press release when research results are published. |  |
| 30 | t the institute where I worked, I frequently wrote a public communication (e.g. report, factsheet, policy brief) on my research findings aimed at policy or practice, separately from peer-reviewed scientific publication. |  |
| 31 | It was easy to discuss any problems regarding my research with my co-workers. |  |
| 32 | The institute where I worked actively stimulated informal interaction between co-workers. |  |
| 33 | Generally, I had enough time to sit down and write a good manuscript. |  |
| 34 | I experience high work pressure. |  |
| 35 | I needed to report innovative and novel conclusions in my scientific work to obtain new funding. |  |
| 36 | At the institute where I worked, funders made requests regarding the phrasing of conclusions and messages of my manuscripts. |  |
| 37 | At the institute where I worked, I adapted my discussion or conclusion upon request of the funding agency at least once. |  |
| 38 | When writing my manuscript, I felt I need to take the position of my funder into account. |  |
|  | Do you have any additional comments regarding the above statements? | [open answer] |
|  |  |  |
| **Research Environment Level** | **The following section is about research environment characteristics. Please indicate your agreement with the following statements. This section contains the last two pages with statements.** |  |
|  |  |  |
| 1 | When I frame my conclusions more excitingly, I receive more citations. |  |
| 2 | To make a career in science, I need to demonstrate societal impact. |  |
| 3 | I sometimes approach journalists or other media to achieve public exposure of my results. |  |
| 4 | Usually, I summarize my conclusions in social media such as twitter, LinkedIn or Facebook. |  |
| 5 | My publications should generate media attention. |  |
| 6 | Exciting conclusions will generate media attention. |  |
| 7 | Journalists have exaggerated conclusions of my study in a public communication (i.e. press releases, news items) at least once. |  |
| 8 | There is a lot of competition for better research positions in my field. |  |
| 9 | My publication track record is essential to compete for better research positions. |  |
| 10 | I feel pressure to publish my research in high impact journals. |  |
| 11 | Generally, I try to publish in journals with the highest impact factors. |  |
| 12 | I experience a high publication pressure. |  |
| 13 | Without publication pressure, my scientific output would be of higher quality. |  |
| 14 | I suspect that for some co-workers, publication pressure leads to inappropriate (e.g. exaggerated) conclusions or messages. |  |
|  | Do you have any comments regarding the above statements? | [open answer] |
|  |  |  |
| 15 | Journals will not accept my manuscript unless I frame 'exciting' conclusions or messages. |  |
| 16 | It has happened that in the editing process at a journal, parts of the discussion or conclusions in my manuscript were significantly changed by the editor. |  |
| 17 | My manuscripts would not have been accepted if I would not have emphasized 'positive' findings. |  |
| 18 | When writing my manuscripts I always use a reporting checklist (e.g. STROBE, CONSORT), also when the journal does not require one. |  |
| 19 | The word limit of journals hampers me from writing a good discussion and conclusions section in my manuscripts. |  |
| 20 | I have experienced that one of my co-authors had a conflict of interest with regard to the research findings. |  |
| 21 | I have experienced that a co-author pressured me to write conclusions that suited their own practice. |  |
| 22 | I have experienced that there was a disturbing conflict between co-authors about the content of a manuscript. |  |
| 23 | My publications are of better quality if fewer co-authors are involved. |  |
| 24 | My publications are of better quality if co-authors from other institutes are involved. |  |
| 25 | In the design of my study, I frequently involve stakeholders such as patients or professionals (not funders). |  |
| 26 | I always discuss my preliminary findings and conclusions with stakeholders such as patients or professionals (not funders). |  |
|  | Do you have any additional comments regarding the above statements? | [open answer] |
|  |  |  |
| **General information** | What is your current (research) position? | [list of positions] |
|  | Please specify, |  |
|  | How many years had you been working as a researcher in January 2018? |  |
|  | What was your research position in January 2016 | [list of positions] |
|  | Please specify, |  |
|  | In which academic background were you trained? | 1 = Social Sciences 2 = Epidemiology 3 = (Health) economics 4 = Other |
|  | Please specify, |  |
|  | What is your area of expertise? | [open question] |
|  | How many articles have you currently co-authored in peer reviewed journals? | 1 = 1 2= 2-5 3= 6-10 […..] |
|  | What is your age? |  |
| **End of survey** |  |  |
