# Supplementary material for: Individual, institutional, and scientific environment factors associated with questionable research practices in the reporting of messages and conclusions in scientific health services research publications
Source: BMC Health Serv Res. 2020 Sep 3;20:828. doi: 10.1186/s12913-020-05624-5 (PMC7469341; doi:10.1186/s12913-020-05624-5)
Supplement: Supplementary file 4 — Additional file 4. [file 12913_2020_5624_MOESM4_ESM.docx]

**Supplementarty material 4**

**Additional information on the exploratory factor analyses**

This document describes the methods and results of the exploratory factor analyses conducted to identify factor domains from the survey ‘Publication Practices in Health Services Research’.

**Methods**

The survey ‘Publication Practices in Health Services Research’ contains three domains: the individual domain existing of 18 items, the institutional domain existing of 34 items, and the environmental domain existing of 26 items. We applied an exploratory factor analysis using principal component analysis as extracting method and direct oblimin as rotation method. We used these methods because factors were unlikely to be fully independent from each other. An average score for each factor was used to allow for an equal ‘weighting’ among the factors in the statistical analysis. Items with a load factor 0.3 or above were assigned into a specific factor. Items with multiple load factors were assigned to the most fitting factor based on the largest value of load factors on this specific item. A specific label was given to each particular factor. Based on the researchers’ judgement, items which did not fit the assigned factor were removed. A reliability analyses was performed.

From the exploratory factor analysis we identified 6 factors for the individual domain, 7 factors for institutional domain, and 7 factors for the environmental domain. We conducted a reliability analysis for each factor by using Cronbach’s alpha. Items with a crohnbachs alpha over 0.3 were considered for the bivariate analyses.

**Results**

Table S2.1. describes the factors resulting from the exploratory factor analyses. Table S2.2 describes the reliability of the resulting factors. Two items that strongly decreased the reliability of their assigned factors were removed. Four items that were catogorized together by the factor analyses did not relate well conceptually i.e. items ‘Individual_3, Individual_18, Environment_two_8, and Environment_two_6. These items were therefore considered as single-item factors.

**Table S2.1. Domains identified from the exploratory factor analysis.**

| **DOMAIN** | **FACTOR** | **ITEM NUMBER** | **FACTOR LOADING** | **ITEM DESCRIPTION** |
| --- | --- | --- | --- | --- |
| **Individual** | Ambition in science | Individual_1 | 0.679 | I want to advance my career in science. |
|  |  | Individual_2 | 0.574 | To advance my career, it is important to produce many scientific publications in international peer-reviewed journals. |
|  |  | Individual_4 | 0.640 | It is important that my co-workers have a high opinion of my work. |
|  |  | Individual_8 | 0.476 | I am disappointed if my results are statistically not significant. |
|  |  |  |  |  |
|  | Pressure to create societal impact | Individual_3 | - | To advance my career, it is important to have societal impact through e.g. television interviews, media exposure. |
|  | Contribution to science | Individual_18 | - | My peer-reviewed publications make an important contribution to my scientific field. |
|  |  |  |  |  |
|  | Self-efficacy | Individual_5 | 0.658 | When my last manuscript was accepted by a peer-reviewed journal I was extremely happy. |
|  |  | Individual_14 | 0.640 | I have difficulty communicating with my co-authors about the contents of a manuscript. |
|  |  | Individual_15 | 0.758 | I get nervous when I receive feedback about my manuscript from my co-authors. |
|  |  |  |  |  |
|  | Perception of received training | Individual_7 | 0.546 | I hope to be surprised by my results. |
|  |  | Individual_10 | 0.617 | I have received sufficient training in writing scientific publications. |
|  |  | Individual_13 | 0.775 | I have received sufficient training in research integrity. |
|  |  |  |  |  |
|  | Confidence in writing | Individual_9 | 0.345 | I am sufficiently trained in the research methods I apply. |
|  |  | Individual_11 | 0.623 | I find it difficult to write conclusions based on my research findings for peer-reviewed publications. |
|  |  | Individual_12 | 0.628 | I find it difficult to write recommendations for policy or practice in peer-reviewed publications. |
|  |  |  |  |  |
| **Institutional** | Specific training in reporting messages and conclusions | InstitutionalOne_1 | 0.699 | The institute where I worked provided training (opportunities) regarding the writing of discussion and conclusion sections specifically. |
|  |  | InstitutionalOne_2 | 0.767 | The institute where I worked provided sufficient training on presenting in writing or verbally my findings in lay terms. |
|  |  |  |  |  |
|  | Competitiveness | InstitutionalOne_3 | 0.758 | The institute where I worked rewards high quality publications. |
|  |  | InstitutionalOne_4 | 0.791 | The institute where I worked rewards high numbers of publications. |
|  |  | InstitutionalTwo_6 | 0.663 | There is a strong culture of competitiveness within the department/institute where I worked. |
|  |  |  |  |  |
|  | Data storage | InstitutionalOne_9 | 0.492 | I received training on the formal quality assurance policy regarding the conduct of research at the institute where I worked. |
|  |  | InstitutionalThree_2 | 0.750 | The institute where I worked has strict rules on the storage of data and filing of research material. |
|  |  |  |  |  |
|  | Feedback culture at institute | InstitutionalTwo_1 | 0.882 | I received feedback on my manuscript from my co-workers (not co-authors). |
|  |  | InstitutionalTwo_2 | 0.842 | I find it helpful to receive feedback on my manuscript from my co-workers. |
|  |  | InstitutionalTwo_3 | 0.807 | It was mandatory to discuss manuscripts during formal peer-review groups at the institute where I worked. |
|  |  | InstitutionalTwo_4 | 0.829 | In formal peer-review groups, suggestions were made for the revision in the discussion and conclusion sections of manuscripts. |
|  |  | InstitutionalTwo_10 | 0.684 | At the institute where I worked, I always discussed my manuscripts in a voluntary peer-review group. |
|  |  | InstitutionalTwo_5 | 0.408 | It was compulsory to address the review comments received in the peer-review group. |
|  |  |  |  |  |
|  | Social support | InstitutionalTwo_8 | 0.415 | At the institute where I worked, ideas for new research studies (proposals) are discussed amongst colleagues. |
|  |  | InstitutionalThree_5 | 0.575 | It was easy to discuss any problems regarding my research with my co-workers. |
|  |  | InstitutionalThree_6 | 0.727 | The institute where I worked actively stimulated informal interaction between co-workers. |
|  |  | InstitutionalThree_7 | 0.778 | Generally, I had enough time to sit down and write a good manuscript. |
|  |  | InstitutionalThree_8 | 0.387 | I experience high work pressure. |
|  |  |  |  |  |
|  | Media policy | InstitutionalThree_3 | 0.690 | It is common for the institute where I worked to write a press release when research results are published. |
|  |  | InstitutionalThree_4 | 0.698 | At the institute where I worked, I frequently wrote a public communication (e.g. report, factsheet, policy brief) on my research findings aimed at policy or practice, separately from peer-reviewed scientific publication. |
|  |  | InstitutionalThree_9 | 0.308 | I needed to report innovative and novel conclusions in my scientific work to obtain new funding. |
|  |  |  |  |  |
|  | Influence of funders | InstitutionalThree_10 | 0.751 | At the institute where I worked, funders made requests regarding the phrasing of conclusions and messages of my manuscripts. |
|  |  | InstitutionalThree_11 | 0.813 | At the institute where I worked, I adapted my discussion or conclusion upon request of the funding agency at least once. |
|  |  | InstitutionalThree_12 | 0.809 | When writing my manuscript, I felt I need to take the position of my funder into account. |
|  |  |  |  |  |
| **Environment** | Creating exciting conclusion | ResearchEnvironment_1 | 0.797 | When I frame my conclusions more excitingly, I receive more citations. |
|  |  | EnvironmentTwo_1 | 0.736 | Journals will not accept my manuscript unless I frame 'exciting' conclusions or messages. |
|  |  |  |  |  |
|  |  |  |  |  |
|  | Media contact | ResearchEnvironment_3 | 0.601 | I sometimes approach journalists or other media to achieve public exposure of my results. |
|  |  | ResearchEnvironment_4 | 0.799 | Usually, I summarize my conclusions in social media such as twitter, LinkedIn or Facebook. |
|  |  | ResearchEnvironment_5 | 0.496 | My publications should generate media attention. |
|  |  | ResearchEnvironment_7 | 0.632 | Journalists have exaggerated conclusions of my study in a public communication (i.e. press releases, news items) at least once. |
|  |  |  |  |  |
|  | Pressure from scientific culture | ResearchEnvironment_8 | 0.781 | There is a lot of competition for better research positions in my field. |
|  |  | ResearchEnvironment_9 | 0.627 | My publication track record is essential to compete for better research positions. |
|  |  | ResearchEnvironment_10 | 0.853 | I feel pressure to publish my research in high impact journals. |
|  |  | ResearchEnvironment_11 | 0.717 | Generally, I try to publish in journals with the highest impact factors. |
|  |  | ResearchEnvironment_12 | 0.783 | I experience a high publication pressure. |
|  |  |  |  |  |
|  | Suspicion of co-workers | ResearchEnvironment_13 | 0.599 | Without publication pressure, my scientific output would be of higher quality. |
|  |  | ResearchEnvironment_14 | 0.516 | I suspect that for some co-workers, publication pressure leads to inappropriate (e.g. exaggerated) conclusions or messages. |
|  |  | EnvironmentTwo_7 | 0.807 | I have experienced that a co-author pressured me to write conclusions that suited their own practice. |
|  |  | EnvironmentTwo_9 | 0.752 | My publications are of better quality if fewer co-authors are involved. |
|  |  |  |  |  |
|  | Journal practice | EnvironmentTwo_2 | 0.572 | It has happened that in the editing process at a journal, parts of the discussion or conclusions in my manuscript were significantly changed by the editor. |
|  |  | EnvironmentTwo_5 | 0.757 | The word limit of journals hampers me from writing a good discussion and conclusions section in my manuscripts. |
|  |  |  |  |  |
|  | Stakeholder influence | EnvironmentTwo_11 | 0.784 | In the design of my study, I frequently involve stakeholders such as patients or professionals (not funders). |
|  |  | EnvironmentTwo_12 | 0.879 | I always discuss my preliminary findings and conclusions with stakeholders such as patients or professionals (not funders). |
|  | Co-author conflict of interest | EnvironmentTwo_6 | - | I have experienced that one of my co-authors had a conflict of interest with regard to the research findings. |
|  | Disturbing conflict between co-authors | EnvironmentTwo_8 | - | I have experienced that there was a disturbing conflict between co-authors about the content of a manuscript. |

**Table S2.2. Factors score and the results from reliability analysis**

| **Factors** | **Domains** | **Mean** | **SD** | **Cronbach's alpha** |
| --- | --- | --- | --- | --- |
|  |  |  |  |  |
| **Individual** | Ambition in science | 3.53 | 0.52 | 0.46 |
|  | Self-efficacy | 2.36 | 0.59 | 0.57 |
|  | Perception of received training | 3.66 | 0.57 | 0.51 |
|  | Confidence in writing | 2.85 | 0.57 | 0.42 |
|  | Pressure to create social impact | 3.41 | 0.78 | n.a |
|  | Perception of contribution to science | 3.71 | 0.69 | n.a |
|  |  |  |  |  |
| **Institution** | Specific training | 3.19 | 0.95 | 0.73 |
|  | Competitiveness | 3.40 | 0.90 | 0.73 |
|  | Data storage | 3.47 | 0.86 | 0.37 |
|  | Feedback culture at institute | 3.17 | 0.91 | 0.85 |
|  | Social support | 3.68 | 0.55 | 0.52 |
|  | Media policy | 2.68 | 0.79 | 0.54 |
|  | Influence of funders | 1.74 | 0.73 | 0.79 |
|  |  |  |  |  |
| **Environment** | Creating exciting conclusion | 2.69 | 0.67 | 0.52 |
|  | Media contact | 2.46 | 0.78 | 0.63 |
|  | Pressure from scientific culture | 3.78 | 0.66 | 0.82 |
|  | Suspicions of co-workers | 2.39 | 0.66 | 0.69 |
|  | Journal practice | 2.28 | 0.70 | 0.34 |
|  | Stakeholder influence | 3.13 | 1.01 | 0.71 |
|  | Co-author conflict of interest | 1.87 | 0.84 | n.a |
|  | Conflict between co-author | 2.08 | 1.01 | n.a |
|  |  |  |  |  |

*n.a: not applicable, single item question*
