# Supplementary material for: Individual, institutional, and scientific environment factors associated with questionable research practices in the reporting of messages and conclusions in scientific health services research publications
Source: BMC Health Serv Res. 2020 Sep 3;20:828. doi: 10.1186/s12913-020-05624-5 (PMC7469341; doi:10.1186/s12913-020-05624-5)
Supplement: Supplementary file 5 — Additional file 5. [file 12913_2020_5624_MOESM5_ESM.docx]

**Supplementary material 5**

**Additional information on the results of the stratification between first and last authors**

In this document the bivariate analyses and multivariate models for the stratification between first and last authors are provided.

**Table S4.1 Bivariate analysis between factors from individual, institutional, and scientific environment domain with number of QRPs using Poisson regression (first and last author)**

| **Domain** | **Factors** | **First author** | | | | | **Last author** | | | | |
| --- | --- | --- | --- | --- | --- | --- | --- | --- | --- | --- | --- |
|  |  | **B** | **SE** | **p-value** | **Exp(B)** | **95% CI** | **B** | **SE** | **p-value** | **Exp(B)** | **95% CI** |
|  |  |  |  |  |  |  |  |  |  |  |  |
| **Individual** | Ambition in science | 0.141 | 0.116 | 0.223 | 1.15 | 0.92-1.45 | -0.025 | 0.145 | 0.862 | 0.98 | 0.73-1.30 |
|  | Self-efficacy | -0.060 | 0.100 | 0.552 | 0.94 | 0.77-1.15 | -0.381 | 0.139 | 0.006 | 0.68 | 0.52-0.90 |
|  | Perception of received training | -0.173 | 0.105 | 0.099 | 0.84 | 0.69-1.03 | 0.223 | 0.140 | 0.112 | 1.25 | 0.95-1.64 |
|  | Confidence in writing | -0.008 | 0.110 | 0.992 | 0.99 | 0.82-1.23 | -0.268 | 0.151 | 0.077 | 0.76 | 0.57-1.03 |
|  | Pressure to create societal impact | 0.315 | 0.089 | 0.001 | 1.37 | 1.15-1.63 | 0.256 | 0.093 | 0.006 | 1.29 | 1.08-1.55 |
|  | Perception of contribution to science. | 0.112 | 0.095 | 0.240 | 1.12 | 0.93-1.35 | 0.071 | 0.108 | 0.512 | 1.07 | 0.87-1.32 |
|  |  |  |  |  |  |  |  |  |  |  |  |
| **Institution** | Specific training in reporting messages and conclusions | -0.199 | 0.059 | 0.001 | 0.82 | 0.73-0.92 | -0.108 | 0.082 | 0.189 | 0.90 | 0.76-1.05 |
|  | Competitiveness | 0.054 | 0.074 | 0.463 | 1.06 | 0.91-1.22 | 0.119 | 0.080 | 0.139 | 1.13 | 0.96-1.32 |
|  | Data storage | -0.097 | 0.068 | 0.154 | 0.91 | 0.79-1.03 | 0.032 | 0.089 | 0.722 | 1.03 | 0.87-1.23 |
|  | Feedback culture at institute | 0.117 | 0.069 | 0.091 | 1.13 | 0.98-1.28 | -0.090 | 0.076 | 0.240 | 0.91 | 0.78-1.06 |
|  | Social support | -0.046 | 0.103 | 0.653 | 0.95 | 0.78-1.17 | -0.020 | 0.142 | 0.887 | 0.98 | 0.74-1.29 |
|  | Media policy | 0.090 | 0.095 | 0.346 | 1.09 | 0.91-1.32 | -0.038 | 0.075 | 0.612 | 0.96 | 0.83-1.16 |
|  | Influence of funders | -0.159 | 0.085 | 0.061 | 0.85 | 0.72-1.01 | 0.084 | 0.110 | 0.449 | 1.09 | 0.88-1.35 |
|  |  |  |  |  |  |  |  |  |  |  |  |
| **Environment** | Creating exciting conclusion | 0.280 | 0.120 | 0.020 | 1.32 | 1.04-1.68 | -0.111 | 0.087 | 0.203 | 0.89 | 0.75-1.06 |
|  | Media contact | 0.005 | 0.082 | 0.954 | 1.01 | 0.86-1.18 | -0.090 | 0.098 | 0.360 | 0.91 | 0.76-1.11 |
|  | Pressure from scientific culture | 0.028 | 0.105 | 0.789 | 1.03 | 0.84-1.26 | 0.047 | 0.099 | 0.634 | 1.05 | 0.86-1.27 |
|  | Suspicions of co-workers | -0.058 | 0.087 | 0.507 | 0.95 | 0.79-1.12 | -0.182 | 0.124 | 0.143 | 0.83 | 0.65-1.06 |
|  | Journal practice | -0.107 | 0.087 | 0.221 | 0.90 | 0.76-1.07 | -0.063 | 0.105 | 0.550 | 0.94 | 0.76-1.15 |
|  | Stakeholder influence | 0.107 | 0.061 | 0.080 | 1.11 | 0.98-1.25 | 0.196 | 0.073 | 0.007 | 1.22 | 1.05-1.40 |
|  | Co-author conflict of interest | -0.024 | 0.0794 | 0.758 | 0.98 | 0.84-1.14 | -0.207 | 0.085 | 0.016 | 0.81 | 0.68-0.96 |
|  | Conflict between co-authors | -0.015 | 0.0611 | 0.801 | 0.98 | 0.87-1.11 | -0.152 | 0.066 | 0.023 | 0.86 | 0.75-0.98 |
|  |  |  |  |  |  |  |  |  |  |  |  |

**Table S4.2 Multivariate analysis between factors from individual, institutional, and scientific environment domains with number of QRPs using Poisson regression (first author).**

|  | **Model 1^a^** | | | **Model 2^b^** | | | **Model 3^a^** | | | **Model 4^b^** | | | |
| --- | --- | --- | --- | --- | --- | --- | --- | --- | --- | --- | --- | --- | --- |
|  | **B** | **Exp(B)** | **95% CI** | **B** | **Exp(B)** | **95% CI** | **B** | **Exp(B)** | **95% CI** | **B** | **Exp(B)** | **95% CI** |  |
|  |  |  |  |  |  |  |  |  |  |  |  |  |  |
| Intercept | 0.016 | 1.02 | 0.15-7.09 | 1.180 | 3.25 | 1.10-9.62 | 0.248 | 1.28 | 0.18-0.93 | 1.261 | 3.53 | 1.06-11.67 |  |
| Journal impact factor | - | - | - | - | - | - | -0.114 | 0.89 | 0.80-1.01 | -0.076 | 0.93 | 0.84-1.02 |  |
| Working duration | - | - | - | - | - | - | 0.004 | 1.01 | 0.98-1.03 | 0.012 | 1.01 | 0.99-1.03 |  |
|  |  |  |  |  |  |  |  |  |  |  |  |  |  |
| **Individual** |  |  |  |  |  |  |  |  |  |  |  |  |  |
| Ambition in science | 0.271 | 1.31 | 0.99-1.72 | - | - | - | 0.306 | 1.36 | 0.99-1.85 | - | - | - |  |
| Perception of received training | -0.142 | 0.87 | 0.68-1.11 | - | - | - | -0.139 | 0.87 | 0.68-1.14 | - | - | - |  |
| Pressure to create societal impact | 0.257 | 1.29 | 1.07-1.56 | 0.279 | 1.32 | 1.11-1.58 | 0.216 | 1.24 | 1.02-1.51 | 0.247 | 1.28 | 1.07-1.54 |  |
| Perception of contribution to science. | 0.139 | 1.20 | 0.93-1.53 | - | - | - | 0.192 | 1.21 | 0.94-1.55 | - | - | - |  |
|  |  |  |  |  |  |  |  |  |  |  |  |  |  |
| **Institution** |  |  |  |  |  |  |  |  |  |  |  |  |  |
| Specific training | -0.185 | 0.83 | 0.71-0.96 | -0.165 | 0.85 | 0.75-0.96 | -0.170 | 0.84 | 0.72-0.98 | -0.174 | 0.84 | 0.74-0.96 |  |
| Data storage | -0.018 | 0.98 | 0.82-1.17 | - | - | - | -0.112 | 0.89 | 0.73-1.10 | - | - | - |  |
| Feedback culture | 0.158 | 1.17 | 1.01-1.36 | - | - | - | 0.218 | 1.24 | 1.05-1.47 | - | - | - |  |
| Influence of funders | -0.188 | 0.83 | 0.69-0.99 | - | - | - | -0.173 | 0.84 | 0.70-1.02 | - | - | - |  |
|  |  |  |  |  |  |  |  |  |  |  |  |  |  |
| E**nvironment** |  |  |  |  |  |  |  |  |  |  |  |  |  |
| Creating exciting conclusion | 0.075 | 1.08 | 0.81-1.44 | 0.054 | 1.06 | 0.81-1.37 | 0.060 | 1.06 | 0.79-1.44 | 0.087 | 1.09 | 0.84-1.42 |  |
| Journal practice | -0.090 | 0.91 | 0.76-1.10 | - | - | - | -0.075 | 0.93 | 0.77-1.12 | - | - | - |  |
| Stakeholder influence | 0.060 | 1.06 | 0.92-1.23 | - | - | - | 0.052 | 1.05 | 0.90-1.24 | - | - | - |  |
|  |  |  |  |  |  |  |  |  |  |  |  |  |  |

^a^Included domains with p-value <0.30 in bivariate analysis; ^b^Included domains with p-value <0.05 in bivariate analysis;

**Table S4.3. Multivariate analysis between factors from individual, institutional, and scientific environment domains with number of QRPs using Poisson regression (last author).**

|  | **Model 1^a^** | | | **Model 2^b^** | | | **Model 3^a^** | | | | **Model 4^b^** | | | |  |
| --- | --- | --- | --- | --- | --- | --- | --- | --- | --- | --- | --- | --- | --- | --- | --- |
|  | **B** | **Exp(B)** | **95% CI** | **B** | **Exp(B)** | **95% CI** | | **B** | **Exp(B)** | **95% CI** | | **B** | **Exp(B)** | **95% CI** | |
|  |  |  |  |  |  |  | |  |  |  | |  |  |  | |
| Intercept | 2.201 | 9.03 | 1.54-52.89 | 2.034 | 7.65 | 2.48-23.57 | | 2.212 | 9.13 | 1.47-56.60 | | 2.132 | 8.43 | 2.56-27.74 | |
| Journal impact factor | - | - | - | - | - | - | | -0.066 | 0.94 | 0.86-1.02 | | -0.066 | 0.94 | 0.87-.1.01 | |
| Working duration | - | - | - | - | - | - | | 0.005 | 1.01 | 0.98-1.02 | | 0.004 | 1.01 | 0.98-1.02 | |
|  |  |  |  |  |  |  | |  |  |  | |  |  |  | |
| **Individual** |  |  |  |  |  |  | |  |  |  | |  |  |  | |
| Self-efficacy | -0.190 | 0.83 | 0.59-1.16 | -0.304 | 0.74 | 0.56-0.98 | | -0.198 | 0.82 | 0.59-1.15 | | -0.308 | 0.74 | 0.55-0.98 | |
| Perception of received training | 0.266 | 1.30 | 0.96-1.77 | - | - | - | | 0.253 | 1.29 | 0.95-1.75 | | - | - | - | |
| Confidence in writing | -0.359 | 0.78 | 0.48-1.01 | - | - | - | | -0.360 | 0.70 | 0.48-1.01 | | - | - | - | |
| Pressure to create societal impact | 0.215 | 1.24 | 0.93-1.65 | 0.249 | 1.28 | 1.01-1.62 | | 0.175 | 1.19 | 0.90-1.58 | | 0.173 | 1.19 | 0.93-1.52 | |
|  |  |  |  |  |  |  | |  |  |  | |  |  |  | |
| **Institution** |  |  |  |  |  |  | |  |  |  | |  |  |  | |
| Specific training | -0.114 | 0.89 | 0.74-1.08 | - | - | - | | -0.058 | 0.94 | 0.77-1.16 | | - | - | - | |
| Competitiveness | 0.111 | 1.12 | 0.89-1.40 | - | - | - | | 0.099 | 1.10 | 0.88-1.39 | | - | - | - | |
| Feedback culture | -0.068 | 0.93 | 0.77-1.13 | - | - | - | | -0.094 | 0.91 | 0.75-1.10 | | - | - | - | |
|  |  |  |  |  |  |  | |  |  |  | |  |  |  | |
| E**nvironment** |  |  |  |  |  |  | |  |  |  | |  |  |  | |
| Creating exciting conclusion | 0.003 | 1.01 | 0.78-1.28 | - | - | - | | -0.052 | 0.95 | 0.73-1.24 | | - | - | - | |
| Suspicion of co-workers | -0.031 | 0.97 | 0.72-1.31 | - | - | - | | 0.047 | 1.05 | 0.76-1.44 | | - | - | - | |
| Stakeholder influence | -0.043 | 0.96 | 0.75-1.22 | -0.014 | 0.98 | 0.80-1.21 | | -0.012 | 0.98 | 0.77-1.27 | | 0.037 | 1.04 | 0.84-1.29 | |
| Co-author conflict of interest | -0.112 | 0.89 | 0.74-1.08 | 0.159 | 0.85 | 0.71-1.03 | | -0.083 | 0.92 | 0.75-1.12 | | -0.122 | 0.88 | 0.73-1.08 | |
| Conflict between co-authors | -0.108 | 0.90 | 0.75-1.07 | -0.036 | 0.96 | 0.82-1.13 | | -0.118 | 0.89 | 0.74-1.06 | | -0.052 | 0.95 | 0.81-1.12 | |
|  |  |  |  |  |  |  | |  |  |  | |  |  |  | |

^a^Included domains with p-value <0.30 in bivariate analysis; ^b^Included domains with p-value <0.05 in bivariate analysis;
